# The Story of the Hospitals

**Published:** 1887-08-20

**Authors:** 


### THE STORY OF THE HOSPITALS.

*(Continued from p. 276, vol. ii.)*

### The Royal National Hospital for Consumption, Ventnor.

**The Prevalence of Consumption.** Consumption is a disease which appears to be confined to no particular race of men, but to be the common bane of every nation, civilised or uncivilised. Certain countries have had immunity from this disease claimed for them, but it has generally been found, upon closer examination, that such is not the case. Some nations, however, are its victims to a much larger extent than others, and our own country is unhappily one of the severest sufferers. Not inaptly, consumption has been styled the "Plague of England." It is impossible to walk about our large centres of population, such as London, Birmingham, and Manchester, without being struck by the frequency of the signs of our "national disease" upon the faces especially of the poorer classes. Consumption, in the widest application of the term, signifies a wasting away of the flesh, a premature and gradual decay, but it is more specifically applied to the disease which we know as *phthisis pulmonalis*, or pulmonary consumption—a disease which slowly and insidiously destroys the lungs. A deposit of tubercle takes place and gradually extends itself, eating away the structure of the lung, thus preventing that organ from accomplishing its all-important function. In some rare instances the tubercular deposit will apparently cease its growth, and the harm stops ; but in the vast multitude of cases the disease goes on, and, gaining headway, we have what is known as rapid consumption. In such cases complete cure is hopeless, and the odds in the great struggle 'twixt life and death can but be upon one side. There are some institutions which are open to the reception of advanced cases, but the majority of our consumption hospitals only receive patients who are in the incipient or early stages of the disease, when, in other words, there is at least a hopeful possibility of recovery.

The Ventnor Hospital.

The Royal National Hospital for Consumption and Diseases of the Chest, at Ventnor, was founded for the treatment of this disease in its earlier and more curable form. Ventnor is a town which must be too familiar to my readers to need description. The hospital stands in the lovely and far-famed Undercliff, on the St. Lawrence or Western Road, some three-quarters of a mile from the town. The sea, of which it commands some beautiful views, is three hundred yards away. The visitor cannot fail to be struck by the outward appearance of the institution. The project of its erection originated with Dr. Arthur Hill Hassall, the distinguished analyst, still one of its consulting physicians, and the plan of its structure was devised by him. You might imagine, as you come upon the hospital, that you were viewing a row of marine villas, for the structure is built upon what is known as the separate or cottage principle. The grounds, over twenty acres in extent, are kept in beautiful order. As we stand outside we count ten blocks of houses, in the centre of which is a chapel, where such of the patients as choose attend divine worship on the Sabbath. Others, however, who are not members of the Church of England, are at liberty to go to other places of worship at Ventnor.

An Extension Needed.

The original plan of the hospital, which has been accomplished, comprised the erection of sixteen semi-detached houses, the eight blocks to accommodate about one hundred patients. The first two houses were opened in 1869, and the others have been subsequently added, as the funds of the institution or the munificence of friends has permitted. The provision thus made proved inadequate to the constantly increasing demands made for admission. A ninth block was added, increasing the beds to 118, but these were all too soon absorbed. Now, a tenth block has been erected, and will, it is hoped, be ready for the reception of patients next October. This will raise the number of beds to 138. The Duke of Cambridge, who takes a very warm interest in our medical charities, presided at the annual festival dinner, which took place in the Whitehall Salon of the Hôtel Métropole on 19th April, when his Royal Highness made a strong appeal on behalf of the fund for completing and furnishing the new block.

The Interior of the Hospital.

The interior of the hospital—I should rather say, the interior of the *home*—cannot fail to make a very favourable impression in regard to its exquisite cleanliness, order, and beauty. Each patient (who is required to pay ten shillings a week towards his or her maintenance) has a separate sleeping-room. The advantages of this system are obvious, for it not only secures privacy to the patient, but prevents to a great extent his being annoyed or irritated by the coughs and unrest of other sufferers. A consumption hospital is very different in its requirements from hospitals devoted to the treatment of other diseases. The patient—unless the disease has reached its last stage—is not bedridden. He can do a great deal for himself, wash and dress himself, and even put his room in order. There is, therefore, no need of the " ward" system, with the constant attention of the nurse. Moreover, the protracted character of this disease, making a long stay indispensable, is far better met by this plan. The bedrooms are large and excellently ventilated. The windows admit an abundance of light and pure air. These, with exercise and good food, are, after all, the great curatives of this disease, which is pre-eminently one where *nature* can do much and *art* little.

Character of its Arrangements.

The internal arrangements of the hospital appear to be very complete. The rooms and corridors in the winter are kept at an equable temperature ; each block has one or more bath-rooms, and the kitchens are centrally situated. A subway connects the whole of the houses and the chapel, so that the patients can walk to the dining-halls and to church without going outside the building. The rooms are well and neatly furnished, and not overcrowded. Those of the patients who are fond of reading have access to a library of upwards of 1,200 volumes, while other amusements, in the shape of pianos, harmoniums, billiards, bagatelle, chess, draughts, etc., are provided. The lawn in front is used for croquet and American bowls ; entertainments, etc., are also occasionally given in the large hall, which measures 75 feet by 28 feet.

Its Work.

My description of the hospital has, I fear, been too brief to afford a really adequate idea of the work it carries on. Since the erection of the first block, in the winter of 1869, about 7,000 persons from all parts of England and Scotland have sought relief here, and the greater number have obtained it. Though situated at Ventnor, the charity has strong claims to be considered metropolitan as well as cosmopolitan, for about 50 per cent. of the patients treated come from London. Its offices, also, are at Craven Street, Strand.

